# The baseline interferon signature predicts disease severity over the subsequent 5 years in systemic lupus erythematosus

**DOI:** 10.1186/s13075-021-02414-0

**Published:** 2021-01-16

**Authors:** Lloyd Mai, Arundip Asaduzzaman, Babak Noamani, Paul R. Fortin, Dafna D. Gladman, Zahi Touma, Murray B. Urowitz, Joan Wither

**Affiliations:** 1 Division of Rheumatology, Schroeder Arthritis Institute, University Health Network, Department of Medicine, Faculty of Medicine, University of Toronto, Toronto, Canada; 2 Division of Genetics and Development, Krembil Research Institute, University Health Network, Toronto, Canada; 3 Division of Rheumatology, Department of Medicine, Centre de recherche du CHU de Québec – Université Laval, Quebec City, QC Canada; 4 University of Toronto Lupus Clinic, Centre for Prognosis Studies in the Rheumatic Diseases, Toronto Western Hospital, University Health Network, Toronto, Canada; 5 Department of Immunology, Faculty of Medicine, University of Toronto, Toronto, Canada; 6 Schroeder Arthritis Institute, Krembil Research Institute, 5KD402, 60 Leonard Avenue, Toronto, ON M5T 2S8 Canada

**Keywords:** Systemic lupus erythematosus, Interferon, Disease course, SLEDAI-2K

## Abstract

**Objectives:**

Type I interferons (IFNs) play an important role in the pathophysiology of systemic lupus erythematosus (SLE). While cross-sectional data suggest an association between IFN-induced gene expression and SLE disease activity, interest in this as a biomarker of flare has been tempered by a lack of fluctuation with disease activity in the majority of patients. This led us to question whether IFN-induced gene expression might instead be a biomarker of overall disease severity, with patients with high levels spending more time in an active disease state.

**Methods:**

Levels of five interferon-responsive genes were measured in the whole peripheral blood at baseline visit for 137 SLE patients subsequently followed for 5 years. Log transformed values were summed to yield a composite IFN5 score, and the correlation with various disease outcomes examined. Receiver operator characteristic analyses were performed for outcomes of interest. Kaplan-Meier curves were generated to compare the proportion of flare-free patients with high and low IFN5 scores over time.

**Results:**

The baseline IFN5 score was positively correlated with the adjusted mean SLE disease activity index-2000, number of flares, adjusted mean prednisone dose, and number of new immunosuppressive medications over the subsequent 5 years. Optimal cut-offs for the IFN5 score were determined using Youden’s index and predicted more severe outcomes with 57–67% accuracy. A high baseline IFN5 level was associated with a significantly increased risk of subsequent flare.

**Conclusions:**

Measurement of the type I IFN signature is a useful tool for predicting the subsequent disease activity course.

## Background

One of the most characteristic immunologic abnormalities associated with systemic lupus erythematosus (SLE) is elevation of type I interferons (IFNs) [1, 2]. Although these elevations have been difficult to detect in the blood using conventional ELISAs, a significant proportion of SLE patients have elevated levels of IFN-induced gene expression in their peripheral blood cells, suggesting that some of these cells have transited an environment with increased elaboration of type I IFN. In cross-sectional studies of SLE, elevated levels of IFN-induced gene expression, typically measured as scores incorporating expression of a subset of IFN-induced genes, were shown to correlate with disease activity [3–6]. Although these initial observations raised the possibility that the IFN signature might be a useful biomarker to predict imminent changes in disease activity, subsequent longitudinal studies performed by ourselves and others found that IFN-induced gene expression, as measured by a subset of IFN-induced genes, did not fluctuate contemporaneously with disease activity [6, 7]. More recently, it has been argued that the subset of IFN-induced genes typically used to measure the IFN signature in these studies are not the IFN-induced genes that are most responsive to changes in disease activity [8, 9]. However, there remains some controversy regarding this point, since even the genes reported to be more variable over time have not been shown to consistently correlate with disease activity in longitudinal studies [10].

Despite the lack of correlation between the IFN signature and disease activity in some previous studies, we previously showed that patients with high levels of IFN-induced gene expression were more likely to have had a recent flare of their disease and had a higher rate of subsequent flare [6]. These observations, together with a recent report showing that SLE patients in remission with high serum IFN-α levels, as measured by an ultrasensitive technique (single molecule array assay), have a higher rate of subsequent flare than those with normal levels [11], led us to hypothesize that the IFN signature might be a biomarker of overall disease severity and that the correlation between this signature and disease activity in cross-sectional studies is due to these patients spending more of their time in an active disease state. In this study, we addressed this question through examination of the correlation between baseline IFN signature with disease activity and treatment over the subsequent 5 years.

## Methods

### Subjects and data collection

One hundred and thirty-seven SLE patients satisfying 4 or more of the revised 1997 American College of Rheumatology (ACR) classification criteria for SLE [12] or 3 ACR criteria plus a typical histological lesion of SLE on renal or skin biopsy were recruited through the University of Toronto Lupus Clinic at the Centre for Prognosis Studies in the Rheumatic Diseases. All patients had a minimum of 5-year prospective follow-up through the clinic. The clinic typically follows patients at 2- to 6-month intervals regardless of disease activity, with clinical and laboratory information being collected using a standardized protocol at each assessment.

### Clinical measures

Disease activity was determined using the SLE disease activity index-2000 (SLEDAI-2K) [13]. Clinical SLEDAI-2K (cSLEDAI-2K) scores were calculated by omitting the contribution of hypocomplementemia and anti-dsDNA positivity to the total score. A flare was defined as a change in the cSLEDAI-2K ≥ 1 that prompted an escalation in therapy (initiation or increase in the dose of corticosteroids and/or addition of an immunosuppressive medication). Severe lupus flares were defined based on the SELENA-SLEDAI Flare Index [14, 15]. Disease damage was calculated using the Systemic Lupus International Collaborating Clinics (SLICC)/ACR damage index (SDI) [16] and disease activity over time was evaluated by calculating the adjusted mean cSLEDAI-2K (cAMS) [17]. The failure to achieve a lupus low disease activity state (LLDAS), as previously defined [18], on at least 50% of visits or ≥ 2 flares occurring in the course of 5-year follow-up was considered to represent a severe activity course. Patients were defined as having serologically active clinically quiescent (SACQ) or serologically quiescent clinical quiescent (SQCQ) lupus if they had at least a 2-year period without clinical activity (cSLEDAI-2K = 0), with or without persistent serologic activity (i.e., positive anti-dsDNA antibody and/or hypocomplementemia), respectively [19]. Both SACQ and SQCQ patients could be taking anti-malarials, but not corticosteroids or immunosuppressive medications. For patients who were inactive at baseline, time to first flare (any or severe) was calculated by subtracting the date at the baseline visit from that of the first flare visit, and for patients who were active at the first visit, by subtracting the date of the first visit showing clinical improvement as determined by reduction in the SLEDAI from that of the first subsequent flare visit.

### Measurement of IFN-induced gene expression

For measurement of interferon (IFN)-induced gene expression, total RNA was isolated from the whole peripheral blood archived in PAXgene tubes (Applied Biosystems) and gene expression was quantified by NanoString using a custom array (nanoString Technologies), as previously described [20]. Log_2_ normalized expression levels of 5 IFN-induced ubiquitously expressed genes (*EPSTI1*, *IFI44L*, *LY6E*, *OAS3*, *RSAD2*) were then summed to generate a composite IFN5 score [21].

### Statistical analysis

For comparisons of differences between two groups, the Mann-Whiney *U* test was performed. The significance of the association between variables was determined using Spearman’s correlation coefficient. Kaplan-Meier curves were generated to plot the proportion of flare-free patients over time and the significance of the difference between curves determined using the Gehan-Breslow-Wilcoxon test. A receiver operating characteristic (ROC) analysis was performed to evaluate the ability of the IFN5 score to discriminate between various disease outcomes, and the optimal IFN5 scores for predicting each of these outcomes were determined using Youden’s index. All statistical analyses were performed using GraphPad software (La Jolla, CA, USA).

## Results

### IFN-induced gene expression correlates with markers of disease severity at baseline

Demographic, clinical, and treatment information at the initial baseline visit for the 137 SLE patients are shown in Table 1. The majority of patients were recruited at the time of their regular follow-up visits in the clinic (mean disease duration ~ 10 years). Eighty-five (62%) of the patients had active SLE at the time of recruitment (cSLEDAI-2K ≥ 1), of whom ~ 60% had active nephritis, as determined by the presence of proteinuria ± the other renal components of the SLEDAI-2K. Type I IFN-induced gene expression was quantified by summing log_2_ transformed normalized expression levels of 5 ubiquitously expressed IFN-induced genes (IFN5 score), as in our previous studies [20, 21]. IFN5 scores in the SLE patients ranged from 38.3 to 75.3, with a mean of 61.9. As previously observed by ourselves and others, at the baseline visit, there was a moderate positive correlation between the IFN5 score and the SLEDAI (*ρ* = 0.334, *p* < 0.0001) and a moderate negative correlation with age (*ρ* = − 0.299, *p* = 0.0004) and disease duration (*ρ* = − 0.242, *p* = 0.0043) [3–6, 22, 23]. Although there was an association between the levels of C3 and dsDNA antibodies and the IFN5 score (*ρ* = − 0.214 and 0.230, *p* = 0.012 and 0.007, respectively), the association with the SLEDAI-2K was not driven solely by these serologic components, since a positive correlation was also seen with the cSLEDAI-2K (*ρ* = 0.308, *p* = 0.0003). When the clinical components of the SLEDAI-2K were examined separately, the IFN5 score was positively associated with the presences of arthritis (*ρ* = 0.224, *p* = 0.0086) and mucocutaneous involvement (*ρ* = 0.209, *p* = 0.014). No association was seen between the IFN5 score and the presence of renal involvement (associations were not examined for descriptors with ≤ 10 patients), nor was there an association between treatment with anti-malarial or immunosuppressive drugs and IFN5 score.

**Table 1:** Demographic and clinical information for the SLE cohort at baseline visit

	All patients, *N* = 137	High IFN5, *N* = 96	Low IFN5, *N* = 41
Age; mean ± SD (range)	36.4 ± 14.1 (18–76.3)	33.6 ± 12.5 (18–66.6)	43.0 ± 15.6 (18–76.3)
Female Sex; *N* (%)	118 (86.1)	83 (86.5)	35 (85.4)
Disease duration (years); mean ± SD (range)	10.8 ± 9.2 (0–48.5)	9.7 ± 8.3 (0–33.3)	13.3 ± 10.6 (0.1–48.5)
SLEDAI-2K; mean ± SD (range)	6.6 ± 6.3 (0–29)	7.4 ± 6.8 (0–29)	4.8 ± 4.8 (0–20)
cSLEDAI-2K; mean ± SD (range)	4.7 ± 5.6 (0–25)	5.3 ± 6.0 (0–25)	3.3 ± 4.2 (0–16)
Clinical features; *N* (%)			
CNS	3 (2.2)	2 (2.1)	1 (2.4)
Vasculitis	4 (2.9)	3 (3.1)	1 (2.4)
Arthritis	17 (13.1)	15 (15.6)	2 (4.9)
Myositis	0 (0)	0 (0)	0 (0)
Nephritis	52 (38.0)	35 (36.4)	17 (41.5)
Rash	21 (15.3)	18 (18.8)	3 (7.3)
Alopecia	22 (16.8)	18 (18.8)	4 (9.8)
Ulcers	13 (9.5)	12 (12.5)	1 (2.4)
Pleuritis	4 (2.9)	3 (3.1)	1 (2.4)
Pericarditis	2 (1.5)	1 (1.0)	1 (2.4)
Low complement	59 (43.1)	42 (43.8)	17 (41.5)
dsDNA Abs	75 (54.7)	61 (63.5)	14 (34.1)
Fever	5 (3.6)	4 (4.2)	1 (2.4)
Thrombocytopenia	4 (2.9)	3 (3.1)	1 (2.4)
Leukopenia	3 (2.2)	3 (3.1)	0 (0)
SDI; mean ± SD (range)	1.4 ± 1.6 (0–8)	1.2 ± 1.6 (0–8)	1.8 ± 1.6 (0–5)
Prednisone dose (mg); mean ± SD (range)	15.9 ± 20.7 (0–150)	16.8 ± 21.9 (0–150)	13.7 ± 17.5 (0–60)
Anti-malarials; *N* (%)	104 (75.9)	73 (76.0)	31 (75.6)
Immunosuppressives; *N* (%)	86 (62.8)	63 (65.6)	23 (56.1)
Azathioprine	35 (25.5)	25 (26.0)	10 (24.4)
Mycophenolate	45 (32.8)	33 (34.4)	12 (29.3)
Methotrexate	7 (5.1)	6 (6.2)	1 (2.4)
Cyclosporine	2 (1.5)	1 (1.0)	1 (2.4)
Cyclophosphamide	2 (1.5)	2 (2.1)	0 (0)
Others	1 (0.7)	1 (1.0)	0 (0)

*Abbreviations*: *SD* standard deviation, *N* number, *SLEDAI-2K* SLE disease activity index-2000, *cSLEDAI-2K* clinical SLEDAI-2K, *SDI* SLE damage index

### Baseline IFN-induced gene expression correlates with disease severity over the subsequent 5 years and predicts subsequent disease activity course

**Fig. 1:**
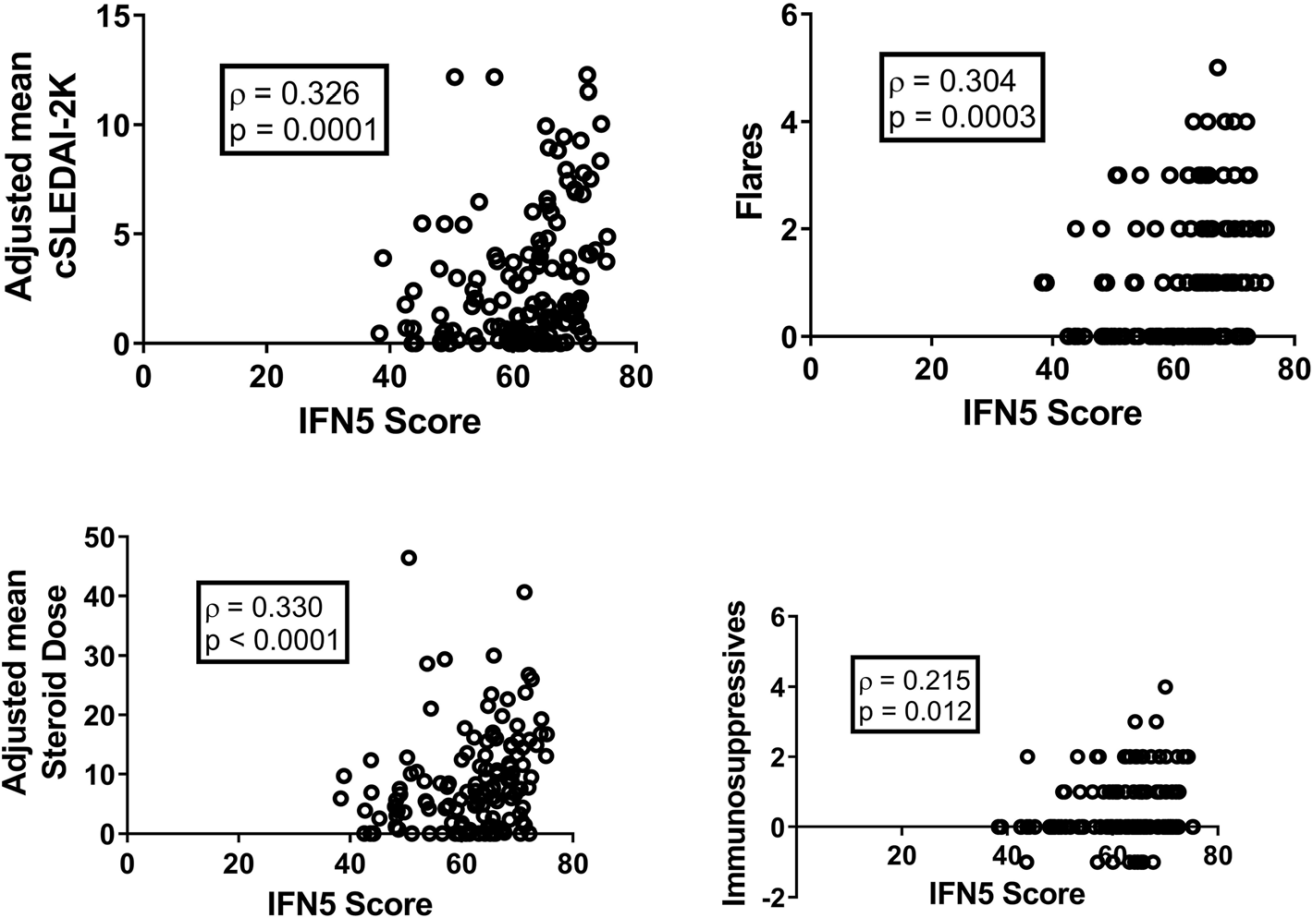
Association between the baseline IFN5 score and disease severity in the subsequent 5 years. Correlation between the IFN5 score and specific disease outcomes. Each open circle represents an individual patient. Boxes show the Spearman correlation coefficient and *p* value for each outcome

We and others have published that the IFN signature, as measured by a subset of IFN-induced genes, remains relatively stable for at least 2 years in the majority of patients despite fluctuations in disease activity [6, 7, 10]. Given this stability, we questioned whether the association between the IFN5 score and disease activity at a single point in time reflects overall increased disease severity (i.e., more time with active disease and/or more flares) in patients with high levels of type I IFN, rather than contemporaneous fluctuations with disease activity in individual patients over time. To address this possibility, we examined whether the IFN5 score at baseline visit correlated with these indicators of disease severity over the subsequent 5 years. As shown in Fig. 1, the IFN5 score at the initial visit was positively correlated with the adjusted mean cSLEDAI-2K (cAMS) and number of subsequent flares over the next 5 years. Not surprisingly, these two indicators of disease severity correlated with each other (*ρ* = 0.626) and also with the adjusted mean corticosteroid dose (*ρ* = 0.720 for cAMS and *ρ* = 0.656 for flares) and the number of new additional immunosuppressive agents used (*ρ* = 0.531 for cAMS and *ρ* = 0.602 for flares) over the next 5 years, and both of these also correlated with the baseline IFN5 score (Fig. 1). No correlation was seen between the IFN5 score and organ damage, as measured by the change in the SDI over the 5-year period, possibly due to the relatively short time period of follow-up in the context of this measurement. Consistent with this possibility, the mean change in the SDI was only 0.4 over this period (range 0–4) with the majority of patients (> 75%) demonstrating no increase in damage.

Previous work suggests that patients who spend a substantial proportion of their time in LLDAS have better outcomes than those with more active disease [18, 24, 25]. Seventy-four patients were in LLDAS at least 50% of the time during the subsequent 5 years. Of these patients, 26 had at least a 2-year period of SACQ or SQCQ, with the remainder having LLDAS on immunosuppressive medications or low-dose prednisone. There was no significant difference in the IFN5 scores between patients who achieved LLDAS at least 50% of the time but who did not have a 2-year SACQ or SQCQ period and those that did (mean IFN5 score LLDAS no SACQ/SQCQ = 58.91 ± 8.55; SACQ/SQCQ = 61.49 ± 7.87, *p* = 0.25). In contrast, baseline IFN5 scores were significantly lower in patients who were in LLDAS at least 50% of the time as compared to those who were not (mean IFN5 score LLDAS present = 59.65 ± 8.30; LLDAS absent = 64.41 ± 8.10, *p* = 0.0002). Taken together, the data indicate an association between the IFN5 score and disease severity over the subsequent 5 years.

**Fig. 2:**
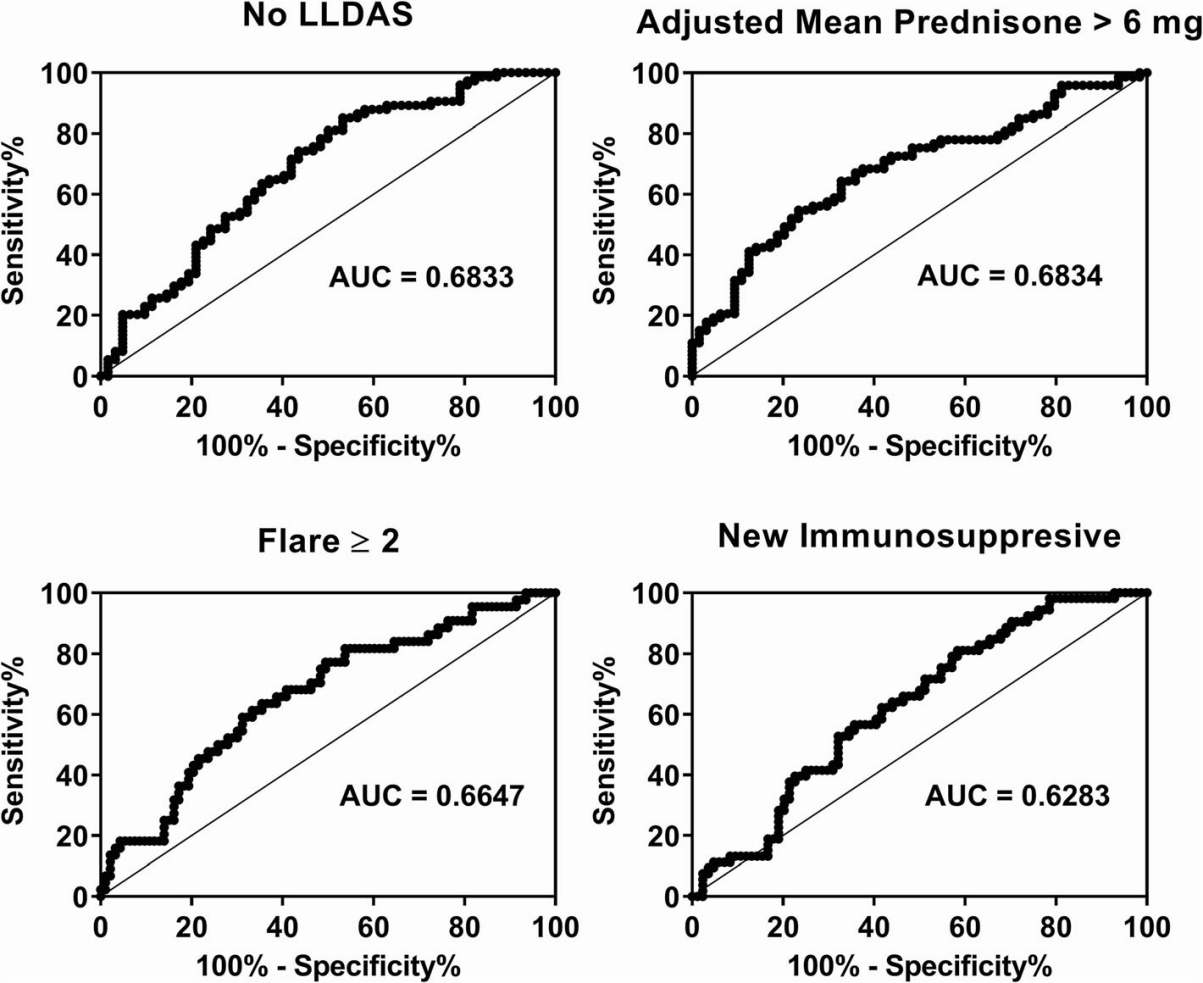
Ability of the baseline IFN5 score to predict disease outcomes over the subsequent 5 years. ROCs are shown for various disease outcomes, with AUCs indicated

To further explore the association between disease severity and baseline IFN5 score, we examined whether baseline IFN5 scores could predict subsequent disease course. Outcomes examined included a persistent high disease activity state, as indicated by the failure to achieve LLDAS at least 50% of the time over the subsequent 5 years, and a requirement for sustained corticosteroid treatment, as indicated by a mean adjusted prednisone dose of > 6 mg. We also examined recurrent flares (≥ 2 subsequent flares during the 5-year follow-up) and the need for introduction of new immunosuppressive agents. Results of the ROC analysis can be seen in Fig. 2, and as shown in Table 2, the optimal IFN5 score for predicting these outcomes varied between 60 and 66.45, with sensitivities ranging from 63.6 to 83.8% and specificities ranging from 41.7 to 67.2%. Using these cut-offs, a high IFN5 score had the greatest ability to discriminate differences in disease activity (66.9% accuracy), followed by corticosteroid treatment (65.7% accuracy) and flares (64.2% accuracy), and was least able to discriminate the need for a new immunosuppressive agent (56.9% accuracy). Notably, patients with the highest IFN5 scores (> 71, *n* = 16) had a 75% chance of being on a mean adjusted prednisone dose ≥ 7.5 mg and an 81.2% chance of being in a sustained high disease activity state with recurrent flares (median = 2) over the subsequent 5-year period. These findings indicate that the baseline IFN5 score can be used to predict with reasonable accuracy disease outcome over the subsequent 5 years.

**Table 2:** Results of the ROC analysis of the ability of IFN5 score to predict various disease outcomes

Outcome	Optimal IFN5 cut-off	Sensitivity	Specificity	PPV	NPV
No LLDAS	66.45	83.8	46.7	71.4	65.3
Adjusted mean prednisone > 6	64.26	64.4	67.2	69.1	62.3
≥ 2 flares	64.79	63.6	64.5	63.6	64.5
New immunosuppressive	60.00	81.3	41.7	46.7	77.8

*Abbreviations*: *LLDAS* lupus low disease activity state, *PPV* positive predictive value, *NPV* negative predictive value

### High levels of IFN-induced gene expression at initial visit are associated with an increased risk of subsequent flare

Given the recently reported association between high levels of serum IFNα and risk of subsequent flare over the next year [11], we questioned whether an elevated IFN5 score at baseline was associated with an increased risk of flare and a decreased time to subsequent flare. Overall, 52.9% of patients had at least one subsequent flare and 38.2% had at least one subsequent severe flare over the 5-year period. Subsequent flare rates were decreased in patients who were not in a flare at the baseline visit (*n* = 87, 41.4% flare and 31.0% severe flare) or who were clinically inactive at the baseline visit (*n* = 52, 36.5% flare and 25% severe flare). The average time to flare was 1.372 years for all flares and 1.470 for severe flares.

**Fig. 3:**
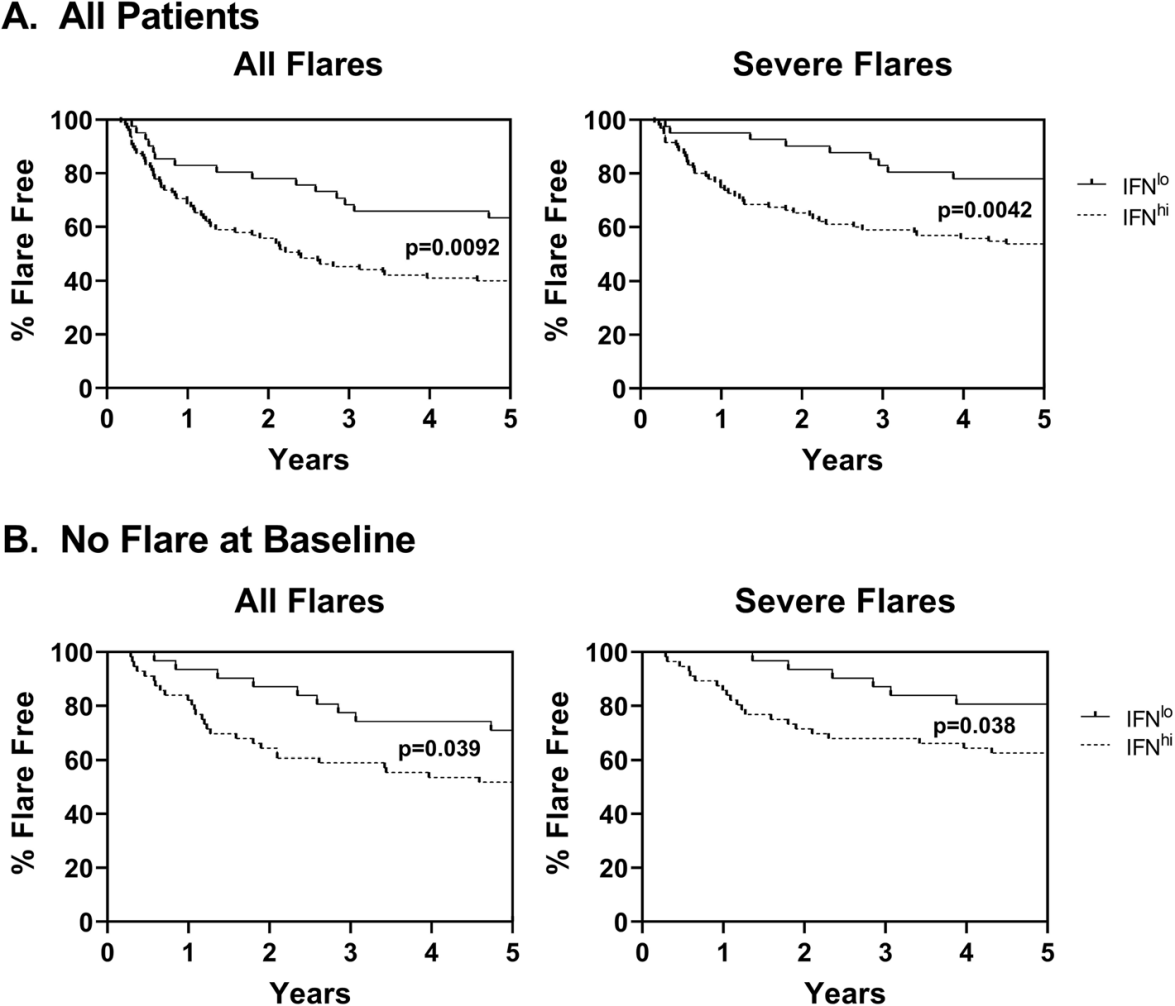
High IFN5 scores are associated with an increased risk of disease flare. Kaplan-Meier curves showing the proportion of flare-free patients over time. The significance of the differences between curves was determined using the Gehan-Breslow-Wilcoxon test

To determine the impact of baseline IFN levels on the risk of subsequent flare, patients were stratified into low and high IFN5 scores based upon whether their IFN5 levels were > 2 SD above the mean of those for healthy controls (*n* = 22, mean = 47.41 ± 6.04). The demographic information for these two groups is shown in Table 1. Although there was significant overlap in the clinical characteristics between groups, patients with low IFN5 scores were older and had a slightly longer disease duration than those with high IFN5 scores. As shown in Fig. 3, there was a significantly increased risk of subsequent flare in patients with high IFN5 scores as compared to those in the normal range, regardless of whether all patients were examined or only those that were not flaring at baseline, with similar results for all flares or just the subset of severe flares. When patients who were clinically inactive at baseline were examined, an increased risk was only seen for all flares (*p* = 0.044). Although there was a trend to a decreased time to flare in patients with high IFN5 scores, this only achieved statistical significance for severe flares in patients who were not flaring at baseline.

## Discussion

In this study, we show that several indicators of disease severity, including the mean clinical disease activity, number of flares, and amount of drug treatment, correlate with the baseline IFN5 score and that a single measurement of this score with appropriate cut-offs can be used to predict with moderate accuracy a more severe disease outcome over the subsequent 5 years. We further show that an elevated baseline IFN5 score is associated with a significantly increased risk of subsequent flare. While it could be argued that this association results from the observation that patients with high IFN5 scores have a higher baseline SLEDAI and that these patients are more likely to have subsequent flares, the same association was seen between IFN5 score and risk of flare in patients who were not flaring or inactive at baseline, suggesting that having a high IFN5 score confers an increased risk of flare independent of baseline disease activity.

Although the IFN5 score was associated with indicators of more severe inflammation, no association with increased organ damage was seen. It is currently unclear whether the 5-year follow-up period was too short for significant damage to develop or whether other genetic and environmental factors play a more important role than IFN in determining the extent of damage that occurs in response to inflammation.

The genes that comprise the IFN5 score are all found within a cluster of genes that has previously been shown to be strongly induced by IFN-α [8, 9], and consistent with this observation, we have previously shown that there is a strong correlation between the levels of serum IFNα, as measured by a high sensitivity ELISA, and the peripheral blood IFN5 score in systemic autoimmune rheumatic disease [20]. Thus, our findings support the concept that measurement of IFN-α levels, either through a single molecule array assay [11] or IFN-induced gene expression, can provide valuable information with regard to the risk of subsequent disease flare. However, there are a couple of notable differences between our findings and those of Mathian et al. [11]. Firstly, our findings suggest that the increased risk of flares extends beyond the first year following measurement, and secondly, the levels of IFN-α, as measured by the IFN5 score, predict not only the risk for flare but the number of subsequent flares.

In SLE, immune complexes containing RNA or DNA have been shown to promote IFN-α production by plasmacytoid dendritic cells through activation of toll-like receptors 7 or 9, respectively [26, 27]. Thus, production of IFN-α is closely associated with the presence of autoantibodies, in particular RNA-associated autoantibodies [3, 4, 20, 28, 29]. The observation that many of these autoantibodies do not fluctuate with disease activity may provide a partial explanation for the lack of association between fluctuations in disease activity and the IFN signature, as measured by the subset of genes that are induced by IFN-α [9], such as those used in the current study. While this lack of association suggests that variations in IFN-α do not directly drive flares of disease, type I IFNs have pleomorphic effects on the immune system, many of which serve to augment the immune dysregulation in SLE [26, 30]. Consequently, the immunologic derangement in patients with higher type I IFN levels may be more severe resulting in a requirement for more aggressive treatment and increasing the likelihood of flares. For example, type I IFNs promote differentiation of T follicular helper and Th1 cells [30, 31], which could enhance generation of autoantibody producing plasma cells and tissue inflammation, respectively, both of which have been associated with disease flares.

The concept that the levels of type I IFN may define overall disease severity rather than contemporaneous flare is supported by studies with type I IFN-targeted therapies. In a recent trial of anifrolumab, an anti-IFN-α receptor antibody that blocks the activity of IFN-α, IFN-β, and IFN-ω, approximately twice as many SLE patients achieved a low disease activity state at week 52 following initiation of treatment as compared to placebo control [32]. Notably, consistent with our findings, within the placebo arm of this study, patients with a high baseline IFN score were significantly less likely to subsequently attain a low disease activity state with conventional SLE therapy over the subsequent 52 weeks. Taken together with our findings, these observations suggest that type I IFN blockade may play an important role in SLE management through prevention/attenuation of subsequent flares of disease and through promotion of a low disease activity state, particularly in difficult-to-treat patients with high baseline IFN-induced gene expression.

A potential limitation of our study is that we examined an unselected prevalent SLE patient cohort of convenience with varying ages and durations of disease. It is possible that at disease inception the levels of IFN-induced gene expression may not be as informative. Studies show that following disease onset patients can follow one of three disease courses, persistent disease activity, recurrent remission and exacerbation, and initial disease activity followed by prolonged disease quiescence [33]. Whether these courses can be predicted by the levels of IFN-induced gene expression at disease inception remains to be seen. In support of this possibility, we have previously shown that patients who have entered a prolonged period of disease quiescence have low levels of IFN-induced gene expression [34], a finding that is recapitulated here. It is currently not known whether these patients had similarly low levels at disease inception. The relative stability of the IFN signature in the majority of patients over at least a 2-year period would argue that this is the case, but it remains possible that changes in the levels of IFN-induced gene expression occur over longer periods of time either as a result of age-related alterations in immune function or as a consequence of active immunoregulation in a subset of patients. These questions will need to be addressed through study of an inception cohort of patients followed longitudinally for many years.

## Conclusions

Measurement of the type I IFN signature at a single time point may be a useful clinical tool for predicting the subsequent disease activity course, with a low IFN5 score bolstering the clinician’s confidence that the patient is likely to have a less active disease course and decreased risk of subsequent flare. In contrast, clinicians should be wary of patients who have very high IFN scores given the high likelihood of more severe disease and may wish to consider more aggressive treatment in this subset of patients.

## Data Availability

All relevant data are contained within the paper.

## Abbreviations

IFN: Interferon
SLE: Systemic lupus erythematosus
ANA: Anti-nuclear antibody
ACR: American College of Rheumatology
SLEDAI-2K: SLE disease activity index-2000
cSLEDAI-2K: Clinical SLEDAI-2K
SDI: Systemic Lupus International Collaborating Clinic (SLECC)/ACR damage index
cAMS: Adjusted mean cSLEDAI-2K
LLDAS: Lupus low disease activity state
SACQ: Serologically active clinically quiescent
SQCQ: Serologically quiescent clinically quiescent
